# Documenting best practices for maintaining access to HIV prevention, care and treatment in an era of shifting immigration policy and discourse

**DOI:** 10.1371/journal.pone.0229291

**Published:** 2020-02-18

**Authors:** Emily A. Arnold, Shannon M. Fuller, Omar Martinez, Julia Lechuga, Wayne T. Steward

**Affiliations:** 1 Department of Medicine, University of California San Francisco, San Francisco, CA, United States of America; 2 Department of Social Work, Temple University, Philadelphia, PA, United States of America; 3 Department of Public Health Sciences, University of Texas El Paso, El Paso, TX, United States of America; Oregon State University, UNITED STATES

## Abstract

Changes in the United States federal-level political landscape have been felt within immigrant communities, and the public health clinics that serve them. We sought to document how HIV prevention and care clinics are reaching and retaining their immigrant community patients during a period of retrenchment of accessible public resources and immigrant rights. From May 2018 through January 2019, we conducted 20 in-depth interviews with clinicians, case workers, advocates, legal experts, and peer navigators in Northern and Central California. Interviews were recorded and transcribed. Several themes emerged which can be grouped into three primary areas: changes post-election, challenges meeting the needs of patients, and best practices for maintaining access to prevention and care services. Post-election, providers reported some of their patients skipping clinic appointments due to fear of Immigration and Customs Enforcement (ICE) raids and deportation while other patients had moved to locations that they felt were less policed. Challenges emerged around linguistic competency, meeting basic needs such as housing stability and employment, and treating mental health sequelae resulting from trauma experienced in home countries or during migration itself. Best practices included hiring bi-lingual and bi-cultural staff, linking to legal services to assist with immigration status, holding trainings around immigrant rights and responses to ICE raids, and building trust with immigrant patients by assuring them that their status would not be collected or reported. In light of adverse policy changes affecting immigrants, agencies have begun to institute best practices to mitigate the negative impact of those policies on their clients and patients.

## Introduction

Social and institutional contexts shape individuals' lives and factors such as employment, housing and living conditions, access to food and social services, and legal status are consequential for well-being. Immigration is a process that is both the result of these factors and can result in changes in each of these areas [1]. Immigration is a complex issue, encompassing a range of individuals and varying migration patterns to the United States (US). Nationally, there are approximately 11.1 million undocumented immigrants living in the US [2]. California is the state that is home to the highest number of undocumented immigrants nationally, hosting approximately 2.4 million individuals. Data show that 8 million Americans live in mixed status families, where at least one household member is undocumented, 72% of these citizens are children [3].

Since the 2016 Presidential campaign, there has been an uptick in anti-immigrant rhetoric and xenophobia in the US. President Donald Trump has regularly criticized immigrants and demanded a border wall to keep out people he characterizes as gang members, terrorists, and criminals [4]. This rhetoric has translated into widespread changes in federal-level immigration policy. Deportations and fears of deportation have both increased [5]. The expansion of immigration enforcement within the interior of the US has led to increases in detention. There was a period in summer 2018 in which border police separated approximately 2600 children from families seeking asylum, with family separations still continuing [6]. There are currently over 13,000 unaccompanied minors in custody [7] and there have been reports of men, women, and children being held separately in overcrowded facilities by the US Border Patrol [8].

Within the US, the federal government has moved to restrict access to a range of social and legal rights and services that are designed to support immigrant families and community members. Examples include significant budget cuts to refugee and resettlement programs, the cancellation of the Deferred Action for Childhood Arrivals Act (DACA) in March 2018, later overturned by the US 9^th^ Circuit Court of Appeals, and changes in the public charge rules that are used to determine granting of certain visas or permanent residency. Changes in the public charge rules have been particularly unsettling for immigrants within the US. Draft changes to the rules were leaked to the public in early 2017, released for public comment in October 2018, and the revised rules were officially published in August 2019. However, nationwide implementation of the rules was blocked by injunction by several courts in October 2019, a move that has been appealed to the US Supreme Court by the Trump Administration. The public charge assessment aims to gauge whether or not an individual may become dependent on government funds–i.e., a “public charge.” Currently, the policy only “counts” cash-based assistance, such as Supplemental Security Income (SSI), in its determination of public charge. However, under the new changes to the rule, other non-cash benefits would also be taken into consideration including comprehensive forms of Medicaid, Supplemental Nutrition Assistance Program (SNAP) (food stamps), Temporary Assistance for Needy Families (TANF), Head Start, Section 8 and other housing subsidies, and institutionalization for long term care, in addition to cash assistance programs [9]. A chilling effect on enrollment in these programs has been noted due to immigrants’ fear of deportation or becoming ineligible for citizenship. For example, there have been 10% decreases in participation in state-level food assistance programs among food insecure immigrant families who have arrived in the past 5 years [10–11]. A systematic study also showed that one in seven adults in immigrant families chose to forego public benefits they were otherwise entitled to in 2018 due to fears related to the proposed changes in public charge [12].

Regulations and funding streams for services can be felt on a visceral level among immigrant community members, directly impacting physical and psychological health and wellbeing, through experiences of minority stress and structural racism [13]. For example, in a cohort of 397 US born children with at least one immigrant parent, fear and worry about the consequences of anti-immigrant policy were associated with higher levels of anxiety, sleep disorders, and raised blood pressure [14]. Anti-immigrant laws can have spillover effects, and impact the health of immigrant communities more generally often due to social and familial ties with those who are at risk for deportation [15–16].

State level policy can mitigate some of the felt effects of federal level changes in the policy landscape. State action has symbolic significance, communicating whether immigrants are welcome regardless of their status [17]. California has positioned itself in opposition to the Trump administration and its anti-immigrant policies, with the California State Senate passing a “sanctuary state” bill on April 3, 2017, and allocating $12 million the same day to provide legal defense to immigrants on the border fighting deportation proceedings. This 2017 California Senate Bill 54, known as the "California Values Act," also prohibits use of state or local resources to assist with federal deportation efforts. California has also extended emergency-basis Medicaid coverage, regardless of immigration status. And as of June 2019, comprehensive Medicaid coverage has been made available to all low-income residents up to age 26. The state allows access to in-state tuition at all of its community and state-level universities, it provides drivers licenses to all residents regardless of immigration status, and it chooses not to formally collect immigration status information in order to protect residents from persecution.

The majority of immigrants to California in the past 15 years hail from countries in Latin America (50%) or the Asia Pacific Island region (40%) [18]. Unfortunately, these immigrant communities are also experiencing disparities along the HIV Care Continuum. In 2017, Hispanics/Latinos made up 46% of new HIV infections in California and they, along with Asian Pacific Islanders, were also less likely to be virally suppressed than Whites [19]. Nationally, only 58% of Latinos living with HIV are virally suppressed, while 63% of Hawaiian/Pacific Islanders and 66% of Asians achieve viral suppression [20]. These communities are disproportionately impacted by HIV, yet experience various barriers to safely accessing HIV prevention, care, and treatment. Indeed, with restrictions on access to public health care, fears of deportation, and anti-immigrant rhetoric increasing, providers have reported that fewer patients are showing up for clinic appointments and renewing their prescriptions [21].

It is within this dynamic environment, where state and federal level policies may be in direct opposition to one another, that we sought to understand how access to prevention, care, and treatment was being maintained for immigrants impacted by HIV and living in California. This inquiry is especially important as HIV intervention strategies are increasingly reliant on biomedical approaches. PrEP and ART treatment require reliable engagement in care and adherence to medication regimens to prevent onward transmission of the virus and ultimately “End the HIV Epidemic” [22]. Indeed, in order to end the HIV epidemic, it is important to focus on barriers to care, including anti-immigration policies which disproportionately impact immigrant populations.

## Methods

From May 2018 through January 2019, we conducted 20 in-depth interviews with providers, case workers, advocates, legal experts, and peer navigators in HIV prevention and care clinics in 3 counties in Northern and Central California. Working with our community collaborators, we developed a list of potential key informants working with immigrant community members and providing HIV-related services in our recruitment sites in California. We reached out to potential informants directly, either in person at professional events or by email to invite them to join the study. We also used snowball sampling and asked enrolled participants to recommend others for us to approach for study participation. Inclusion criteria were being 18 years or older, and being currently on staff at an agency or clinic serving immigrant communities impacted by HIV, and willingness to participate in an in person or phone-based interview with members of the study team. Interviews lasted between 60 and 90 minutes, were conducted by phone or in person, and were audio recorded and transcribed. We asked about current clients, engagement in care, any changes in client experiences in accessing services since January 2017. We also inquired about any practices that the clinics had in place to retain immigrant clients, such as linguistic and cultural competency, access to legal services, and case management and access to wrap-around services and health insurance. Please see a copy of our interview guide provided as Supplemental Information. Informants were offered a $100.00 honorarium in exchange for their time. All informants provided verbal consent, which was audio recorded after the interviewer had reviewed the study information sheet, assessed the informant’s understanding of the study’s risks and benefits, and answered any questions regarding participation. The study protocol was reviewed and approved by the Institutional Review Board at the University of California San Francisco.

Using our guide and an initial review of the transcripts, we followed thematic analysis [23] to capture and organize the data, allowing for the use of both deductive and inductive coding. Deductive codes such as “challenges accessing care” were those that were derived from the interview guide, while inductive codes such as “mix of patient experiences” emerged from the data. A team of analysts completed initial coding on 3 transcripts in order to build coder agreement and refine the codebook, discussing and resolving differences in coding to achieve a 90% agreement threshold. Dr. Arnold and Ms. Fuller then independently coded the remaining transcripts. Comparing cases from excerpts extracted from our entire data set, we were able then to distill our findings and present them below.

## Results

Participants were from diverse ethnic and racial backgrounds, and filled a number of roles within clinical and service agencies. Case managers/social workers/navigators were 35% of the sample, 30% were medical providers, 25% were legal or policy experts, and 10% were clinic administrators. Please see Table 1 for information regarding our sample characteristics. We report data from our informants based on their professional role, in order to protect the anonymity of our participants.

**Table 1:** Characteristics of participants (N = 20).

	N (%)
**Role**	
Case Manager, Social Worker, Navigator	7 (35%)
Provider (MD, NP, PA)	6 (30%)
Legal / policy expert	5 (25%)
Clinic Administrator	2 (10%)
**Ethnicity**	
Hispanic/Latino(a)	12 (60%)
Non-Hispanic/Latino(a)	8 (35%)
**Race**	
Asian	4 (20%)
American Indian or Alaska Native	2 (10%)
Black / African American	1 (5%)
Native Hawaiian / Pacific Islander	1 (5%)
White	6 (30%)
Other	4 (20%)
Refuse to Answer	2 (10%)

Themes can be distilled into 3 primary areas: changes in the immigration climate following the 2016 presidential election, challenges in meeting the needs of patients, and best practices for maintaining access to prevention and care services. Please see Table 2 for a summary of our thematic findings and representative quotes from our participants.

**Table 2:** Themes and representative narratives.

Theme	Representative Quote
Changes in the immigration climate following the 2016 presidential election	Following the election, through our medical-legal partnership, we heard that there was one doctor from [a large public hospital] who said that someone had refused emergency medical treatment because they were concerned that they wouldn't be able to pay it back and that would affect their immigration status. (*Legal Expert*) It's a mix of two experiences…The ones that are more established, they come in and tell me that they're scared, and we talk about that and we process it and we talk about resources and supports and all that kind of stuff…For my newer patients who have been undocumented, people who have come in in the last year and a half or so—some of them have actually disappeared. *(Medical Provider)*
Theme 2: Challenges in meeting needs of clients	Housing for undocumented is just plain difficult. Because housing, everything, needs ID. And everything needs Social Security numbers. Everything needs that. So for the undocumented, housing is off. I can't find anything for undocumented clients. *(Case Manager)* I think another big [challenge], honestly, is [finding] mental health services related to anxiety and depression or PTSD or immigration-specific trauma. A lot of either experiences that people are having here because of stress because of their status, or living under threat of deportation. Or experiencing family separation. Or fear of any of the above. And then also extraordinarily traumatic experiences in people's home countries, and extraordinarily traumatic experiences of immigration. (*Administrator*)
Theme 3: Best practices for maintaining access to prevention and care	I guess I would stand back to say it's pretty baked into the mission and the culture of [our clinic] to be a welcoming space irrespective of somebody's immigration status. So, a very large portion of our patient population is undocumented…But I would say the whole registration process is a very judgment-free zone where people just acknowledge what their status is and then we just go about getting them whatever coverage we can…And then we do periodically, like at staff meetings, we've had most recently a presentation about how to talk with patients who are worried about ICE raids and what they're rights are. (*Medical Provider*) I'm an immigrant myself…Basically, I can sympathize with undocumented immigrants. Even though I didn't come in as undocumented, I can understand the difficulty and challenge as to embracing a new culture, a new environment. And basically, things that I didn't know I'm actually helping clients [navigate] now. (*Case manager*) A lot of them are migrant farm workers. They work until 5:00 p.m. or later, so that means that my staff are working later because they will contact the client and establish a time and a place to meet them in their community so that it's easy for them—meeting them at their house or meeting them somewhere, and meeting them after work. Explaining what's going on and why it's important to test. Because staff are phlebotomy trained, they'll do the blood draw and HIV test out in the field. *(Administrator)*

### Theme 1: Changes in the immigration climate following the 2016 presidential election

Post-election, providers reported some of their patients skipping clinic appointments due to fear of Immigration and Customs Enforcement (ICE) raids and deportation. In general, there was a sense of urgency among clients to clarify their immigration status, often motivated by fears of deportation. This included applying for asylum and finalizing name changes among trans-identified patients. There was a great deal of fear and misinformation, which were fueled by rumors that spaces like public transit stations and the clinics themselves were being monitored by ICE.

There's a sense of urgency—even for people who are residents or have permanent residency, but are not U.S. citizens. There's this urgency of like: oh my God, I should get my citizenship … People are scared. With everything they hear on the news every day, if it's not an attack on someone's rights, it's literally like some sort of attack on someone's personal space and emotional and social wellbeing. Even if it's not happening directly to them, it really does seep into your psyche, and I think that there becomes this community level of anxiety and a heightened sense of urgency. *(Program Manager)*

Other informants noted that they had patients that had moved to locales where they felt less policed. One case manager explained that, “We have a couple of clients that were here and the rumors about ICE have been here much, so they move up to Nevada and they say they'll stay there for a while until things calm down. We have some that haven't come back.” For others, the levels of continuity of care differed based on how recently the patient had started coming to clinic.

It's a mix of two experiences. One is for my more established patients who know us and have experienced that embracing of them, no matter what their legal status is or whatever their immigration status is. The ones that are more established, they come in and tell me that they're scared, and we talk about that and we process it and we talk about resources and supports and all that kind of stuff…For my newer patients who have been undocumented, people who have come in in the last year and a half or so—some of them have actually disappeared. *(Medical Provider)*

Another provider concurred:

I could say that my undocumented folks who I established care with at least a year or two before all this stuff changed, they're still in care. They're still engaged in care because we've got their cases going. They trust us; they know us. We've been advocating for them around their legal status. The patients who we don't—we haven't had that established [link], they're the ones who are disappearing. To me, there's a clear difference between before and after. (*Medical Provider*)

Legal experts highlighted the impact of the changes in immigration policies and the lack of clarity around the implications of changes to the public charge rules. The ambiguity regarding what would be considered “non-cash benefits” under the proposed public charge rules also raised intense concerns among frontline workers, medical providers, and legal experts.

We don't know what the [new public charge] rule is going to say. But at least the leaked version, I mean housing advocates are getting involved because it would make public housing and subsidized housing a benefit that could be taken into consideration for public charge… So I mean not only is it dealing with medical [benefits], which before was not something that would be taken into account, which obviously has a very direct impact, but it's also housing, food benefits, benefits for your children. (*Legal Expert*)

Here, an attorney shares two stories of immigrants refusing care or services to which they were entitled:

I feel like there probably was that population of people that is not even seeking care that we just don't know about. Not seeking care or testing out of fear of the policies and the effects that they could have. So, for example, following the election, through our medical-legal partnership, we heard that there was one doctor from [a large public hospital] who said that someone had refused emergency medical treatment because they were concerned that they wouldn't be able to pay it back and that would affect their immigration status…I remember a phone call I got from a client. She and her husband had applied for new visas, they were undocumented, but her children were U.S. citizens and she basically asked if she should disenroll them from Medi-Cal [California’s Medicaid program]. (*Legal Expert*)

Another legal expert summed it up nicely, stating, “The rumors of those different public charge things are just as bad and scary as actually having promulgated anything.”

### Theme 2: Challenges in meeting needs of clients

According to informants, clients were already experiencing challenges common among vulnerable individuals living with HIV, such as maintaining housing and food security, access to substance use and mental health services, HIV-related stigma and, for some, homophobia and/or transphobia. However, these challenges were exacerbated by other factors specific to immigrants. For example, navigating an unfamiliar health care system, language barriers, fears related to accessing public services due to proposed changes in public charge rules, and lack of funding for social services for immigrant clients made it especially challenging to meet needs of immigrant clients in a holistic way.

Housing for undocumented is just plain difficult. Because housing, everything, needs ID. And everything needs Social Security numbers. Everything needs that. So for the undocumented, housing is off. I can't find anything for undocumented clients. If it's already hard for documented clients, the undocumented clients just become another level of no access to housing. *(Case Manager)*

The majority of [our clients] are MSM [men who have sex with men], trans, or LGBT [lesbian, gay, bisexual, transgender], and I think there's already a lot of shame around their sexual gender identity. And I think there's this piece of like, I'm undocumented. I'm an immigrant. What do I have a right to? Am I causing more problems by asking for support and asking for help? *(Program Manager)*

A lot of these [health care] systems, they technically…have quote-unquote "translating services that are automated" that they call. There's a line that they call, and then there's someone there on the phone to translate. And then through this phone that translation happens….And that's not accessible. There's scheduling problems, so if there's no one on-site, and especially for positive folks with things that are happening live, then there's this lag time…It's already hard enough even if you do speak the language to disclose and what to say and who to trust. So there's all these layers. (*Legal Expert*)

In addition, informants pointed to the unmet need for treating mental health sequelae resulting from trauma experienced in home countries or during migration itself.

I think another big [challenge], honestly, is mental health services related to anxiety and depression or PTSD or immigration-specific trauma. A lot of either experiences that people are having here because of stress because of their status, or living under threat of deportation. Or experiencing family separation. Or fear of any of the above. And then also extraordinarily traumatic experiences in people's home countries, and extraordinarily traumatic experiences of immigration. Like of either crossing without papers and all of the things that people have to do to make that happen. (*Program Manager*)

### Theme 3: Best practices for maintaining access to prevention and care

In light of the identified challenges, there was a recognized need to provide comprehensive, culturally competent care. Best practices cited by participants included hiring bi-lingual and bi-cultural staff, including those who are medical providers themselves, linking patients to legal services to assist with immigration status and asylum, holding trainings for both clinic staff and patients around immigrant rights and responses to ICE raids, and building trust with immigrant patients by assuring them that their status would not be collected or reported to the authorities.

One important dimension reported across our sites was the need to strengthen partnerships between legal and medical providers. Here a medical provider talks about his patients expressing a need for legal support:

I've actually had some patients in this new kind of Trump era who have shifted and have actually been more afraid—some of them have sought out lawyers in this last year to try to actually get asylum. Which is a little bit opposite of what you would think. Because sometimes you would think, oh, well, maybe they're so afraid they don't want to see a lawyer. But I actually have a number of patients who were like, hey, I either want to go from permanent resident to citizen. Or I have a few that said, hey, I want to try this asylum thing because I'm just really nervous.

Many of the clinics and institutions we spoke to had developed medical-legal partnerships in direct response to these needs, which were helpful to both the doctors as well as the attorneys involved in them. On a related note, providers also discussed learning about what needed to be documented in their letters to the court when patients were applying for asylum on the basis of their HIV status.

I think when I first started as a physician at—I didn't really know what to write for the lawyer. And there's no guidelines. So that's another thing, like sort of thinking about what do physicians need to know about what they are supposed to write. And I didn't get that much guidance from the lawyer. And actually what helped me was one of the providers who's no longer here, she had shared a letter that she wrote. Like, “Hey, this is what I usually write. I sort of have this similar format, and you're welcome to sort of use mine. It's not the same thing, but just make sure you kind of cover the same key points.” And that was really helpful. So I sort of have the same concept for each letter that I wrote and tailor it to the patient, and so explaining why. You should touch on the diagnosis, explain it a little bit. Usually emphasize that they're on these medicines. If they have to go back to their country, care would be interrupted in this way. I have a number of patients who are on medicines that they couldn't get if they were sent back to their country. So, emphasizing that. If there's any other complexities to their care, why they need to stay here.”(*Medical Provider*)

Cultural competency was also tremendously important. For our informants, this meant a variety of things, including providing language concordant services, hiring staff that were familiar with the immigration process, and recognizing heterogeneity within immigrant communities.

I guess I would stand back to say it's pretty baked into the mission and the culture of [our clinic] to be a welcoming space irrespective of somebody's immigration status. So, a very large portion of our patient population is undocumented…But I would say the whole registration process is a very judgment-free zone where people just acknowledge what their status is and then we just go about getting them whatever coverage we can. And in [our county], we have a [local] county coverage program, which folks can be eligible for. And then we do periodically, like at staff meetings, we've had most recently a presentation about how to talk with patients who are worried about ICE raids and what they're rights are. (*Medical Provider*)

Posting signs and making immigrants feel explicitly welcome was another step that clinics took to help combat the sense of fear that was permeating immigrant communities. However, as one provider pointed out, sometimes there were fears about being too openly hostile to the federal level policy landscape, particularly if the clinic was a Federally Qualified Health Center (FQHC).

Some of our organizations have signage up around "Immigrants welcomed here," that sort of thing. "Immigrants and refugees welcomed here."… I'm wondering why all our clinics haven't done that. Is there some unifying way that we can demonstrate that we are open and welcoming? …I think one of the reasons why our clinic doesn't do that is they're afraid of raising any red flags with the federal government as a FQHC. There's some fear from us, too, because our funding is always being challenged. *(Medical provider)*

In addition to training reception staff to be welcoming, posting signage, and having protocols and referrals in place for clients, informants stressed that staffing programs and clinics with individuals who spoke multiple languages, and had experienced immigration themselves, was also very important. Here, a program manager explains, “*The experience changes when the work is client centered and non-judgmental*. *Having someone*, *who perhaps has been through that process before*, *someone who looks like them*, *someone who speaks their language*, *is really*, *really helpful*.” Another case manager points directly to his personal immigration experience influencing the support he provides to his clients.

I'm a foreign medical graduate, so from my country—I'm an immigrant myself. I graduated from med school in my country, came here through a refugee situation. Basically, I can sympathize with undocumented immigrants. Even though I didn't come in as undocumented, I can understand the difficulty and challenge as to embracing a new culture, a new environment. And basically, things that I didn't know I'm actually helping clients [navigate] now…Like back when I came, I didn't even understand anything about Medi-Cal. I didn't understand about insurance. I didn't understand a single thing. I was on Medi-Cal, but I didn't know where to use it. I didn't know how to go there. And so now looking back, and now that I'm in this role, I'm learning so much that, oh, I can now help my clients faster as to what they should do or what can they do, what options there are so that they can have a life plan.

Clinics that had co-located and culturally tailored programs, were also very successful in retaining patients, through meeting a comprehensive set of needs. One medical provider talked about the mental health care provider at her clinic:

And then we have a really wonderful mental health team at [our clinic] that is accessible to our patients that are [LGBT identified]. So oftentimes people have either mood disorders or emotional distress that has to do with immigration status or experiences, and so we're making those referrals and trying to help people there… there's one therapist in particular that again is bilingual, bicultural, and also lesbian-identified and has sort of taken a special interest in making herself available to our clients. So we can do warm handoffs when possible directly with her.

Several of our informants, particularly those working in rural settings, noted that taking services directly to immigrants helped lower barriers to testing, and linking to care. One administrator talked about providing HIV testing services literally in the field:

A lot of them are migrant farm workers. They work until 5:00 p.m. or later, so that means that my staff are working later because they will contact the client and establish a time and a place to meet them in their community so that it's easy for them—meeting them at their house or meeting them somewhere, and meeting them after work. Explaining what's going on and why it's important to test. Because staff are phlebotomy trained, they'll do the blood draw and HIV test out in the field. *(Administrator)*

These practices were replicated in more urban settings as well, particularly when patients were reluctant to leave their homes because of fears that ICE was monitoring the clinics or the public transit stations that served the clinic neighborhoods.

In that time [when ICE was monitoring the clinic], we tried to call [our patients]. “You can come in.” “No, no, no, I don't want to go. Maybe reschedule my appointment,” “You can come in for medication.” “Oh, no, no, no, I don't want to go.” So…sometimes we have to go to the corner or the [public train] station and give [the medicine] over to them because they don't want to go to the clinic. *(Case Manager)*

We found that clinic staff and providers across our sites were going to extraordinary limits to ensure that their clients and patients could access the prevention and care services they needed.

## Discussion

To our knowledge, this is one of the first studies to document the challenges associated with a more restrictive immigration policy environment and the best practices to promote continued access to HIV prevention and treatment services for those most impacted by the epidemic. Our findings indicate the importance of system level changes to support access to HIV prevention and care-related services in an era of anti-immigration policy initiatives and increased anti-immigrant stigma.

Similar to other studies, our research documented the profound fear and lack of patient engagement in both prevention and care services as result of changes to federal-level immigration policy and increasing anti-immigrant rhetoric [24]. Providers reported losing patients to follow up due to fears related to their immigration status. In some cases, their patients moved away, in other cases patients refused to leave their homes for fear of being subject to deportation and skipped clinic appointments. Although the public charge rules were in draft format at the time of our data collection, patients were concerned about the impact of using public benefits. A lack of comprehensive services, including inaccessible housing, mental health services, and translation assistance compounded the situation further for immigrants living with HIV, making it difficult for clinic staff to meet their needs even if they were engaged in their HIV care.

At the clinic level, we found that training with frontline staff and providers to ensure that patients were aware of their legal rights within medical and clinic facilities was an important component to maintaining access. Moreover, publicizing trainings that were targeted to patients also augmented feelings of comfort and trust in providers and clinic staff. One natural outgrowth of the emphasis on knowing and defending the legal rights of patients was the establishment and in some cases strengthening of existing medical-legal partnerships. This has been shown to be associated with better health outcomes for immigrant patients in the literature, yet there are few models for the best ways to implement such efforts [25–26]. Such recommendations, however, must also be implemented with careful consideration around documenting patient immigration status in order to preserve privacy and alleviate concerns around medical records being used as a basis for legal action, including deportation.

In addition to these structural level changes, several of our findings underscored the importance of relationships between providers, support staff, and patients. One key to developing trust and rapport was an acknowledgement of the complexity that makes up immigrant communities, and an appreciation for how intersectional stigmas may play a role in the hesitancy that patients may experience around accessing care and prevention for HIV. For example, many patients experienced stigma on the basis of their sexual and gender identities, their HIV status, and their immigration status as well. Clinics that openly acknowledged this complexity and tailored their services by hiring people from within the communities they served were successful at welcoming and retaining clients. Establishing trust by providing culturally competent and bilingual care has been associated with retention in care [27–28]. In addition, having co-located counseling available that was designed to be open to sexual and gender minorities allowed patients to feel cared for in these settings and to have multiple needs met in one site. Co-locating and offering integrated care such as patient-centered medical homes has led to better HIV-related health outcomes for people living with HIV [29–30].

Additionally, policy level interventions and context directly impact access to care and prevention particularly when local and state policy may be at odds with federal level policy. In California, there have been explicit advocacy efforts to maintain robust and comprehensive access to the state’s Medicaid program. Eligibility was recently expanded to include undocumented immigrants up to age 26. Having access to health insurance has been linked with better HIV-related health outcomes for undocumented immigrants in other contexts [31]. Within the larger scope of immigration reform, changes in the definition of public charge rules and their interpretation is unknown. However, it is clear that whatever changes may take place, the process has discouraged many immigrants from accessing services which they are legally entitled to, resulting in lower use of publicly-funded services that could help people meet basic needs [12]. Given the changing policy landscape, clinic-based programs that provide education about immigrant legal rights and access to public services, and partner with legal services can support those living with HIV.

Advancing multi-sectoral approaches that address structural determinants of health are necessary. Housing, food security, mental health, access to employment and human dignity are all part of caring for immigrants with HIV and must be taken into consideration, particularly as efforts to dehumanize immigrant community members and remove vital resources are undertaken by those in the federal government. Utilizing state and local resources to attend to other structural factors that drive and perpetuate HIV-related health disparities could help to mitigate the deleterious impact of anti-immigrant rhetoric and policy on HIV intervention efforts.

As a qualitative study with interviews conducted in three counties and focused on access to HIV care and prevention services, our findings may not reflect all practices among clinics serving immigrant communities across California or the US. Furthermore, the best practices described here may be more difficult to implement in states or countries with more restrictive policies around immigrant rights. As noted earlier, California has progressive state-level immigration policy compared to other parts of the US, including Medicaid coverage for all residents up to age 26. Despite these limitations, our findings highlight the benefits of adapting certain best practices at the clinic level to maintain access to HIV prevention and care services for immigrant patients, addressing system-level barriers that may contribute to poor health outcomes for this population.

## Conclusions

We set out to ascertain the degree to which policy changes regarding immigration status were impacting access to HIV prevention and care services in California, a state that has enacted progressive policy measures to safeguard the health of its undocumented immigrant population. Immigrants impacted by HIV face several challenges in accessing care, including encountering language and cultural barriers, housing instability, lack of comprehensive health care coverage, and mental health sequelae of migration itself. Despite these significant challenges, and a federal policy landscape that has promulgated fear regarding accessing public benefits, clinicians and frontline staff have enacted several best practices on the ground to mitigate the larger structural level barriers that immigrants face. Taken together, these measures underscore the health, wellbeing, and human rights of immigrants impacted by HIV.

## Supporting information

S1 File Impact of anti-immigrant stigma and policy on access to HIV prevention and care among immigrant communities affected by HIV in California. (DOCX)
