# Supplementary material for: Documenting best practices for maintaining access to HIV prevention, care and treatment in an era of shifting immigration policy and discourse
Source: PLoS One. 2020 Feb 18;15(2):e0229291. doi: 10.1371/journal.pone.0229291 (PMC7028255; doi:10.1371/journal.pone.0229291)
Supplement: S1 File — (DOCX) [file pone.0229291.s001.docx]

**Impact of Anti-Immigrant Stigma and Policy on Access to HIV Prevention and Care among Immigrant Communities Affected by HIV in California**

**Provider/Staff Interview Guide**

Facesheet

Interview Date: _________________ Interview Time: _________________

Location of Interview: ___________________________________________________________

Interviewer(s):__________________________________________________________________

Participant ID Number: ___________________________________

Special Circumstances:

-----------------------------------------------------------------------------------------------------------------------------------------------------------------------------------------------------------------------------------------------------------------------------------------------------------------------------------------------------------------------------------------------------------------------------------------------------------------------------------------------------------------------------------------------------------------------

*Give participant separate demographics sheet to complete at the end of the interview.*

1. Could you please tell me about your position and role here?
   1. How long have you been in this role?
   2. Could you walk me through what a typical day looks like for you?
2. Tell me a little bit about your agency. What kinds of populations do you serve?
   1. Approximately what proportion of your patients would you estimate are undocumented?
   2. In what capacity do you work with patients/ clients who are undocumented? How do you know that they are undocumented?
   3. What times of day are you open?
   4. What kinds of services do you offer?
3. Could you describe for me how your agency is funded to provide HIV care/ prevention services?
   1. Are you an FQHC?
   2. Do you receive funding through Ryan White?
4. What are some of the challenges that you/your organization has encountered while trying to serve clients who are undocumented?
   1. What do you think are some of the main barriers that individuals who are undocumented face when trying to receive care here? In other care settings?
      1. What kinds of barriers are hardest to address? Easiest? How so?
   2. How well equipped would you say your agency is to address these issues?
      1. What would make your setting better equipped to deal with these issues?
5. What are some strategies that you (or your agency) have found helpful when trying to engage undocumented clients / immigrant community members in care?
   1. Are there any staffing decisions you have made, or any new programs you have developed to support this population?
6. We’ll be talking to patients as well, but I am curious to hear from your perspective – to what extent are you hearing from clients about fears of deportation or concerns around immigration policy?
   1. To what extent, if at all, have you observed any changes in this regard over recent years?
   2. To what extent have you seen or heard of these concerns affecting client retention or care-seeking behaviors at your agency?
   3. IF YES: Without disclosing any names, could you please give an example of a patient for whom this was an issue?
7. What recommendations would you like to pass along to other agencies working with PLWH / prevention clients who are undocumented?
8. Is there anything else you would like to share that you think would be helpful for us to know?
9. Do you have any questions for me?
10. Is there anyone else you would suggest we interview?

**STOP AUDIO-RECORDING**

*Thank interviewee for his/her time and for participating in the study.*

*Give participant the demographics sheet to complete.*

*Give participant the monetary incentive.*

**DEMOGRAPHIC QUESTIONNAIRE**

**1. What is your gender? (Choose one)**

☐ Male

☐ Female

☐ Transgender: Male to Female

☐ Transgender: Female to Male

☐ Refuse to Answer

**2. What is your age?**

☐ 18-24 years old

☐ 25-34 years old

☐ 35-44 years old

☐ 45-54 years old

☐ 55-64 years old

☐ 65-74 years old

☐ 75 years or older

☐ Refuse to Answer

**3. Which of the following best describes your ethnicity? (Choose one)**

☐ Non-Hispanic

☐ Hispanic / Latino

☐ Don't Know

☐ Refuse to Answer

**4. Which of the following best describes your race? (Check all that apply)**

☐ White

☐ Black or African American

☐ Asian

☐ Native Hawaiian / Pacific Islander

☐ American Indian or Alaska Native

☐ Other

☐ Don't Know

☐ Refuse to Answer

**5. What is your profession/occupation? (Choose one)**

☐ Clinic Administrator

☐ Medical Provider (e.g., MD, NP, PA)

☐ Social Worker / Case Manager

☐ Outreach Worker

☐ Other ________________
